# Comparative Analysis of Cadmium Accumulation in Xerophytic Plants: Implications for Species Selection in Phytoremediation

**DOI:** 10.3390/toxics14020135

**Published:** 2026-01-29

**Authors:** Yusufujiang Yusuyin, Aliya Baidourela, Julati Xiaokelati, Huihui Wen, Kahaer Zhayimu, Qian Sun, Guili Sun, Fuxiang Ma

**Affiliations:** 1 College of Resources and Environment, Xinjiang Agricultural University, Urumqi 830052, China; 2 College of Forestry and Landscape Architectural, Xinjiang Agricultural University, Urumqi 830052, China; 3 College of Ecology and Environment, Xinjiang University, Urumqi 830046, China

**Keywords:** arid oasis ecosystems, cadmium accumulation, woody plants, phytoremediation, organ-specific distribution

## Abstract

This study systematically investigates cadmium (Cd) accumulation and translocation mechanisms in woody plants through integrated analysis of 16 species. Roots consistently exhibited the highest Cd concentrations (0.26 ± 0.13 mg/kg), serving as primary accumulation sites, while bark functioned as a critical secondary storage organ (0.22 ± 0.09 mg/kg) with strong physiological coordination to roots (r = 0.72, *p* < 0.001). Leaves demonstrated strict Cd restriction (0.09 ± 0.05 mg/kg) and low variability (CV = 48.7%), indicating evolutionary adaptations to minimize phytotoxicity in photosynthetic tissues. Three functional groups were identified: hyperaccumulators (e.g., *Ulmus pumila*, root/leaf ratio = 6.37), excluders (e.g., *Malus spectabilis*, root/leaf ratio = 1.12), and intermediate species (e.g., Syringa oblata) with balanced translocation patterns. Strong root-bark correlations (r = 0.68) and negative stem-leaf associations (r = −0.42) revealed complex interorgan translocation dynamics. Cd speciation analysis showed dominant residual fractions in soils (60–80%) and elevated water-soluble or weakly bound Cd in roots (35–52%). These findings provide a mechanistic basis for designing species-specific phytoremediation strategies, including phytoextraction and ecological stabilization. It will identify suitable tree species for effectively stabilizing or containing the metal pollution within a defined area, thereby preventing its lateral spread or leaching.

## 1. Introduction

Cadmium (Cd) contamination in arid oasis agroforestry presents increasing ecological and health hazards, primarily due to the extensive use of metal-contaminated irrigation water and the overapplication of phosphate fertilizers [1,2,3]. In contrast to humid regions, the mobility of Cd in arid soils is exacerbated by alkaline pH levels and a deficiency of organic matter, which enhances its bioavailability and facilitates its incorporation into food chains [4]. The efficacy of phytoremediation is limited in water-scarce environments due to the low survival rates of traditional hyperaccumulator species (e.g., *Sedum alfredii* and *Noccaea caerulescens*). The mobility and bioavailability of Cadmium (Cd) are markedly increased in arid regions, attributed to alkaline soils (pH > 8.5) and a low organic matter content (<1.2%). This situation presents a remediation challenge, as traditional hyperaccumulator plants frequently do not thrive in drought conditions [5]. As a result, xerophytic plants, which possess inherent adaptations to aridity and salinity, deserve exploration as potential candidates for the stabilization or extraction of Cd in these difficult environments. The combination of low cadmium leaching and its consequent high bioavailability in arid soils renders these regions particularly vulnerable to the long-term persistence and environmental risk of cadmium contamination: 38% of global croplands affected by cadmium (Cd) are situated in water-limited areas, yet less than 15% of remediation research focuses on these environments [6,7]. The urgency of this issue is exacerbated by rapid urbanization in arid cities such as Urumqi, where industrial emissions and traffic-related non-exhaust emissions intersect with vulnerable ecosystems, resulting in new pathways for contamination [8]. The lack of specialized phytomanagement strategies for these hybrid landscapes not only threatens urban food systems but also accelerates the degradation of ecosystems, with Cd bioavailability in arid urban soils now surpassing World Health Organization (WHO) thresholds by 40–60% [9,10]. It is important to contextualize the potential application of this phytoremediation strategy [11].

The primary objective of this study is to identify xerophytic plant species that can effectively uptake high levels of cadmium (Cd) and stabilize it within their harvestable tissues, thus offering a sustainable method to diminish Cd bioavailability in contaminated arid soils. Xerophytes have evolved various adaptations to cope with metal stress, including rhizosphere acidification and the secretion of metal-binding exudates. Nevertheless, there are still considerable gaps in our understanding regarding the organ-specific distribution of cadmium (Cd) in these plants, which is essential for evaluating both the effectiveness of remediation efforts and the associated ecological risks [12,13,14]. This discrepancy underscores a significant challenge in pinpointing plant species that can successfully manage the extraction, stabilization, and exclusion of Cd, particularly in the face of aridity and human-induced pollution. Existing methodologies frequently lack cohesion, neglecting to consider the spatial variability of contamination hotspots and the specific preferences of different species for metal fractionation. Such oversights lead to ongoing rebound risks and the possibility of unsustainable remediation results [15,16].

The observed interspecific variation in Cd accumulation and partitioning cannot be explained by soil-side bioavailability alone, a factor well-characterized in studies of metal ion speciation and competition [17,18]. This study provides mechanistic insights into the physiological processes subsequent to initial uptake—including differences in root cell wall binding, chelator (e.g., phytochelatin) production.

This research addresses existing gaps through a comprehensive examination of cadmium (Cd) dynamics in Urumqi, a hyper-arid major city that serves as a representative case of the interactions between urbanization and climate change. Our results provide answers to two questions posed in our hypotheses: (1) preliminary findings indicate that various arid-adapted species might influence Cd speciation in unique manners. What are the fundamental physiological or rhizospheric processes that contribute to these species-specific variations in Cd fractionation? (2) What spatial strategies and species combinations can effectively reduce the risks of contamination rebound in gradient areas? These findings bridge the significant gap between ecological adaptation and remediation efficacy, presenting a scalable model applicable to arid urban environments worldwide, where the challenges of climate resilience and pollution management converge.

## 2. Materials and Methods

### 2.1. Setting of Sampling Points

**Figure 1:**
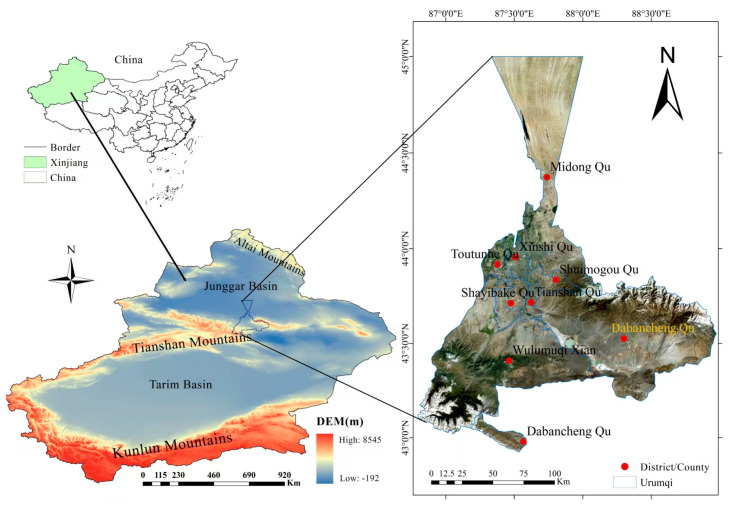
Study area.

The soil samples for this study were collected from Urumqi City northwest China (42.45–44.08° N; 86.37–88.58° E), characterized by a typical mid-temperate, semi-arid continental climate. The climate is dry, with an annual average temperature of 8.3 °C, annual precipitation of 25.8 mm, and annual average sunshine hours of 222.3 [19]. The rapid economic development of Urumqi has recently had a significant impact on the urban soil (Figure 1).

Soil samples were collected along the Yan’erwo (43.71° N, 87.58° E) Interchange towards the Jingxin Expressway (43.72° N, 87.59° E–43.97° N, 87.62° E) direction at the southern, middle, and northern sections of Hetan Expressway (43.79° N, 87.61° E–43.89° N, 87.60° E)as well as the Shuimogou (43.83° N, 87.65° E) Scenic Area (Figure 1). Samples were collected from three representative urban land-use types: (i) public parks and green spaces, (ii) roadside soils, and (iii) residential area soils. Soils in the study area, classified according to the World Reference Base for Soil Resources (WRB), are predominantly strongly anthropogenically modified Urbic Anthrosols and Urbic Cambisols. Through investigation and analysis of the configuration structure of tree species in roadside green belts, representative (high number and high frequency) tree species configuration structures were selected for experimental analysis.

### 2.2. Collection and Treatments of Soil Samples

To ensure the representativeness of soil sampling within each selected urban forest patch (hereafter referred to as a “site”), we established at least three sampling points per site. Soil samples were obtained from the root zone of the selected trees utilizing a soil auger, which was drilled vertically to a depth ranging from 0 to 25 cm. The gathered soil was subsequently separated into two distinct layers: 0 to 10 cm and 10 to 25 cm. The 15 subsamples collected from each site were then thoroughly homogenized. From this mixed material, approximately 1 kg of soil was obtained using the quartering method to form a single composite sample representing that site. Across all 70 investigated sites, this yielded a total of 140 (two soil layers) composite soil samples.

Soil sample processing: The soil samples were air-dried, homogenized by grinding, and sieved through a 100-mesh sieve. Approximately 1.0 g aliquots were digested using a HF-HClO4 method [20] in PTFE crucibles through sequential treatments with 7 mL HF and 7 mL HClO4 (heated to near dryness), repeated HF treatment, followed by addition of 20 mL distilled water and final dissolution in 5 mL 3% HNO_3_. The resulting solutions were diluted to 50 mL, filtered into plastic tubes, and analyzed for heavy metal concentrations by ICP-OES. The average soil pH ranges from 7.5 to 8.8.

The ICP-OES analysis (Agilent 5900, Agilent Technologies, Inc., Santa Clara, CA, USA) coupled with multi-element calibration standards (0.1–100 mg/L) to achieve detection limits in the μg/L range and linear calibration curves (R^2^ > 0.999) for quantitative analysis, with quality control ensured through certified reference materials and periodic wavelength re-calibration to maintain analytical precision. Detailed information was provided in Text S1.

### 2.3. Collection and Processing of Plant Samples

An investigation was conducted on artificially established urban forest (Table 1) species located 5–10 m from the embankment along the Hetan Expressway. Based on the frequency (refers to the percentage of sampling plots in which a given tree species was present) and abundance (refers to the total number of individual trees of a given species counted across all sampling plots) of species distribution, 16 greening tree species were selected for testing, totaling 16 × 3 trees (48 trees in total). The specific test species and corresponding areas are shown in Table 1. Samples of leaves, branches, bark, and roots were collected from the 16 greening tree species. Leaves, branches, and bark were gathered from all directions (east, west, south, north) of each tree and mixed uniformly to form one composite sample. Roots were collected from four directions at two-thirds of the canopy’s vertical projection and mixed to form one composite sample. A total of 16 × 4 × 3 (192) plant samples were obtained.

In Table 1, “/” indicates that the species is a small shrub, metrics such as DBH and crown width are not applicable. Plant sample processing: To ensure representativeness, the plant samples were pre-digested with HNO_3_ overnight at ambient temperature, followed by sequential addition of H_2_O_2_ and HF. The sealed vessels in stainless steel jackets underwent thermal digestion at 150–170 °C for 9 h. The digested solutions were then diluted to 25 mL with 1% HNO_3_ [20], 2015) Chinese National Standard (HJ 491-2019) [21]. The ICP-OES analysis (Agilent 5900, Agilent Technologies, Inc., Santa Clara, CA, USA) coupled with multi-element calibration standards (0.1–100 mg/L) to achieve detection limits in the μg/L range and linear calibration curves (R2 > 0.999) for quantitative analysis, with quality control ensured through certified reference materials and periodic wavelength re-calibration to maintain analytical precision. Detailed information was provided in Text S1.

### 2.4. Classification of Heavy Metal Forms in Samples

The Tessier 5-step extraction method was used to determine the classification of potential trace elements in soil and TSP samples [22,23]. The mass balance of potential trace elements was performed by comparing the total metal content in the samples by single-stage digestion of the sample with the sum of potential trace elements in individual fractions analyzed by sequential extraction. The metal speciation in this study is categorized as: exchangeable form (EF), carbonate form (CF), Fe-Mn bound form (FMF), organic matter bound form (OMBF), and residual form (RF) and the detailed information was provided in Text S2.

### 2.5. Pollution Assessment in the Study Area

The Nemerow comprehensive pollution index was employed to evaluate roadside pollution conditions [24,25,26]. The calculation formula is as follows:

(1) $$P_{i}=\frac{C_{i}}{S_{i}}$$

(2) $$P=\sqrt{\frac{P_{iave}^{2}+P_{i\max}^{2}}{2}}$$

In Equation (1), Pi is the single pollution index of the i-th factor, Ci is the measured concentration value of the i-th factor, and Si is the evaluation standard value of the i-th factor. In Equation (2), Pi ave is the average index value; Pi max is the maximum pollution index of a single pollutant; P is the comprehensive pollution index of the sampling point. This study uses the soil environmental background values of Urumqi City as the reference. Pollution level classification: P ≤ 0.7 is considered clean; 0.7 < P ≤ 1.0 is considered relatively clean; 1.0 < P ≤ 2.0 is considered mild pollution; 2.0 < P ≤ 3.0 is considered moderate pollution.

Calculation of heavy metal enrichment and translocation factors in plants: The Bioconcentration Factor (BCF)] [27,28,29], with the formula as follows:

(3) $$BCF=\frac{C_{i}}{C_{s}}$$

In Equation (3), BCF represents the enrichment coefficient; Ci denotes the content of heavy metal elements in different parts (various organs) of the plant; Cs indicates the content of heavy metal elements in the corresponding rhizosphere soil.

### 2.6. Data Processing and Analysis

This study utilized Excel l (Microsoft 365, version 2404), Origin Pro (2024, version 10.1.0.164), R language (version 4.3.3), and SPSS (IBM SPSS Statistics, version 29.0.1) statistical software to process the experimental data, with all data presented as mean ± standard error. Computational processing was implemented using Python 3.11 with SciPy (v1.10.1) and pandas (v1.5.3) libraries, while graphical visualizations were created using R software (v4.2.2) with ggplot2 (v3.4.2) and ggpubr (v0.4.0) packages [30,31] facilitated through Hiplot Pro (https://hiplot.com.cn/), an integrated web-based platform for biomedical data analysis and visualization. Data presented as mean ± SD. All experimental data were obtained from triplicate measurements and are expressed as the mean ± standard deviation (SD). The choice of specific statistical tests was predicated on prior validation of data distribution and variance homogeneity. For datasets that met both normality and homoscedasticity assumptions, parametric tests were utilized.

Multiple Group Comparisons: A one-way analysis of variance (ANOVA) was conducted. In instances where significant differences were identified (α = 0.05), post hoc pairwise comparisons were performed using Tukey’s Honestly Significant Difference (HSD) test.

Correlation Analysis: The relationships between continuous variables were analyzed using Pearson’s correlation coefficient.

The stability of Ward’s hierarchical clustering was rigorously validated through 1000 bootstrap iterations. Non-parametric analyses (Wilcoxon signed-rank test and Kruskal–Wallis test) were employed to accommodate small sample size requirements.

## 3. Results and Analysis

### 3.1. Integrated Analysis of Cd Accumulation and Translocation Patterns in Woody Plants

**Figure 2:**
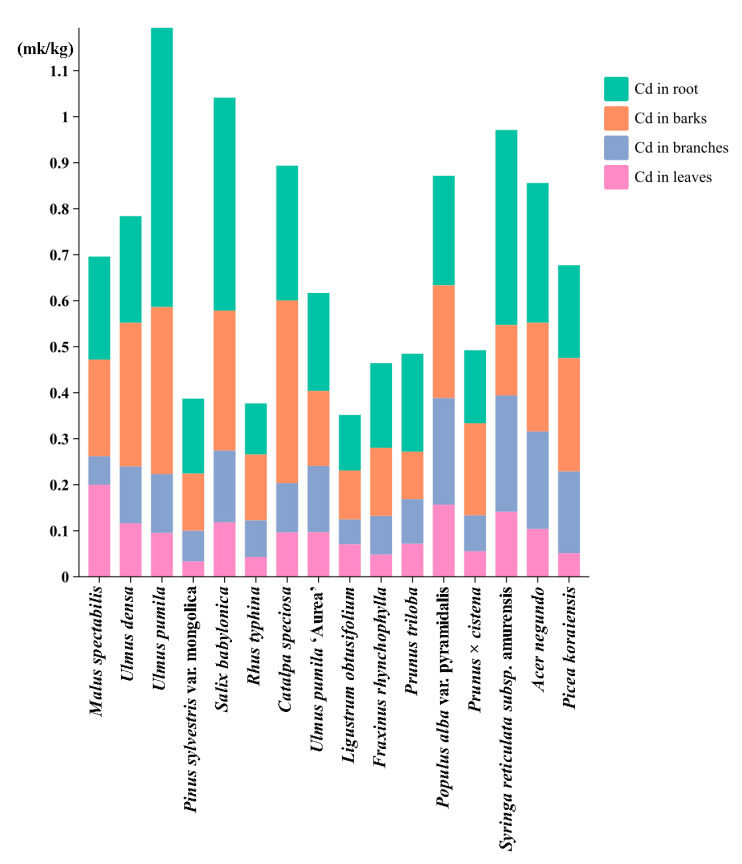
Organ-specific Cd accumulation in plants.

**Figure 3:**
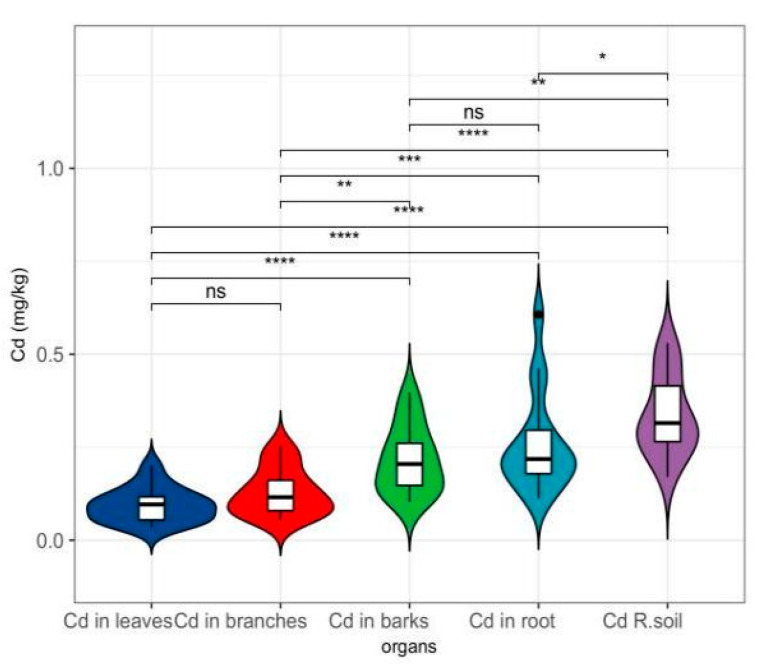
Differential analysis of total Cadmium (Cd) content between plant organs and soil. Note: Data are presented as mean ± SD. Significance levels are indicated as follows: * *p* < 0.05, ** *p* < 0.01, *** *p* < 0.001, **** *p* < 0.0001; ns, not significant.

The study reveals clear organ-specific patterns of cadmium (Cd) accumulation among various tree species (Figure 2 and Figure 3). The roots exhibited the highest concentrations of Cd (0.259 ± 0.134 mg/kg), thereby confirming their function as the primary sites of accumulation. The bark acted as a significant secondary storage organ, sustaining elevated Cd levels (0.216 ± 0.091 mg/kg), likely facilitated by xylem transport. Conversely, the leaves displayed a stringent restriction on Cd accumulation, presenting the lowest concentrations (0.094 ± 0.046 mg/kg) and exhibiting minimal variability (CV = 48.7%), which suggests an evolutionary adaptation aimed at safeguarding photosynthetic tissues (Figure 2 and Figure 3).

Interspecies comparisons highlighted three functional groups with divergent Cd management strategies. *Ulmus pumila* (Dense-crowned Elm) was identified as a hyperaccumulator, exhibiting exceptional root enrichment (root/leaf ratio = 6.37) and pronounced bark/stem partitioning (2.84). Conversely, *Malus spectabilis* (Chinese Flowering Crabapple) adopted an exclusion strategy, maintaining near-equilibrium root-leaf distribution (root/leaf ratio = 1.12) while developing the highest bark/stem ratio (3.38), indicative of efficient Cd sequestration in peripheral tissues. Intermediate species like *Ulmus pumila* showed balanced organ distribution (root/leaf ratio = 2.00), positioning them as reliable bioindicators for pollution monitoring (Figure 2 and Figure 3).

Transport dynamics revealed complex interorgan relationships. The strong root-bark correlation (r = 0.68, *p* < 0.01) supports a coordinated translocation system, while the negative stem-leaf association (r = −0.42) suggests competition for Cd allocation. Notably, the bark/stem ratio’s significant correlation with root/leaf partitioning (r = 0.61, *p* < 0.05) emphasizes bark’s dual role as both a transport conduit and a biological filter, potentially modulating whole-plant Cd distribution (Figure 2 and Figure 3).

These findings directly inform phytoremediation strategies. Hyperaccumulators such as *Ulmus pumila* and *Pinus sylvestris* (root/leaf ratio > 4) are prime candidates for phytoextraction in heavily contaminated soils. Species with moderate translocation efficiency (Syringa oblata; root/leaf ratio 2–3) serve as stabilizers for ecological reconstruction, while the low-translocation phenotype of *Malus spectabilis* provides a natural marker for delineating pollution boundaries. The observed organ-specific accumulation patterns and species-level adaptations collectively advance our understanding of Cd biogeochemistry in woody plants, offering a mechanistic basis for tailored phytomanagement solutions.

**Figure 4:**
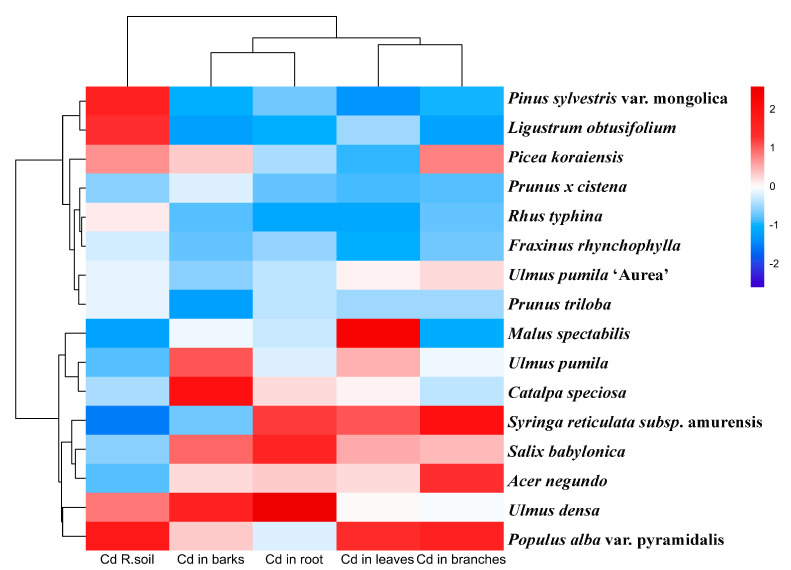
Cluster analysis of Cd content in different plants after normalization.

Kruskal–Wallis tests revealed highly significant differences in pollutant accumulation across plant organs (Figure 3) (H = 29.31, *p* < 0.001), with descending concentrations ranked as roots (0.259 ± 0.134 mg/kg) > bark (0.216 ± 0.091 mg/kg) > branches (0.128 ± 0.063 mg/kg) > leaves (0.094 ± 0.046 mg/kg). Root-dominated accumulation was prominent, exhibiting 2.76-fold higher concentrations than leaves (Cliff’s delta = 0.57, large effect size) and the highest variability (CV = 51.6%), emphasizing its pivotal role in pollution response. Bark demonstrated dual functionality as both accumulator and barrier, showing minimal differentiation from roots (delta = 0.18) but significant divergence from twigs (delta = 0.57), suggesting secondary fixation mechanisms post-xylem transport. Bidirectional translocation constraints were observed, with notably inefficient leaf allocation from branches (delta = 0.35, moderate effect) contrasting with efficient bark-to-twig transfer (delta = 0.57). Strong positive correlation between bark and root concentrations (r = 0.72, *p* < 0.001) contrasted with negative branch-leaf associations (r = −0.42), reflecting divergent source-sink allocation strategies. Notably, leaves exhibited low intraspecific variability (CV = 48.7%), supporting their reliability as pollution biomarkers. Root-to-leaf ratios exceeding 2.5 (mean 2.76) indicated widespread enrichment potential across species, with *Ulmus pumila* demonstrating exceptional hyperaccumulator characteristics (ratio 6.37). These findings systematically quantify phyto-compartmentalization dynamics and provide critical biomarkers for environmental monitoring applications (Figure 3 and Figure 4).

Represented by *Ulmus pumila* (r = 0.607) and *Salix babylonica* (r = 0.463), showing strong positive root-bark Cd correlations (*p* < 0.01), indicative of efficient vascular transport mechanisms. Bark-storage cluster: Featuring *Populus alba* var. pyramidalis (r = 0.245) and *Acer negundo* (r = 0.236), with significant bark-root Cd accumulation associations (r = 0.605). Leaf-restricted cluster: Exemplified by *Pinus sylvestris* (r = −0.368) and *Ligustrum obtusifolium*, demonstrating significant negative correlations between leaf Cd and soil concentrations (Figure 4).

*Ulmus pumila* displayed classic phytoextraction traits (BCFroot = 1.41). Stabilization potential: *Populus alba* showed bark-specific sequestration capacity (0.245 mg/kg), supporting phytostabilization applications.

*Pinus sylvestris* demonstrated exceptional leaf exclusion efficiency (TFleaf = 0.065), qualifying as pollution boundary barrier vegetation. This systematic classification provides critical insights for targeted phytoremediation strategies based on species-specific accumulation profiles.

Root-bark synergistic accumulation exhibited a highly significant positive correlation (r = 0.605, *p* < 0.001), highlighting xylem transport’s pivotal role. Soil-leaf antagonistic effects manifested as significant negative correlations (r = −0.369, *p* < 0.05), suggesting potential protective exclusion mechanisms. Moderate positive branches-root translocation linkages (r = 0.468, *p* < 0.05) reflected secondary xylem allocation strategies.

### 3.2. Interspecific Variation in Cd Speciation Transformation

This section systematically quantifies the stratified distribution of cadmium (Cd) speciation fractions (F1: Exchangeable, F2: Carbonate-bound, F3: Fe-Mn oxide-bound, F4: Organic matter-bound, F5: Residual) across pedological gradients (0–10 cm vs. 10–25 cm horizons) and phyto-compartments (foliar vs. root tissues in *Ulmus* spp. and *Populus* spp.).

**Figure 5:**
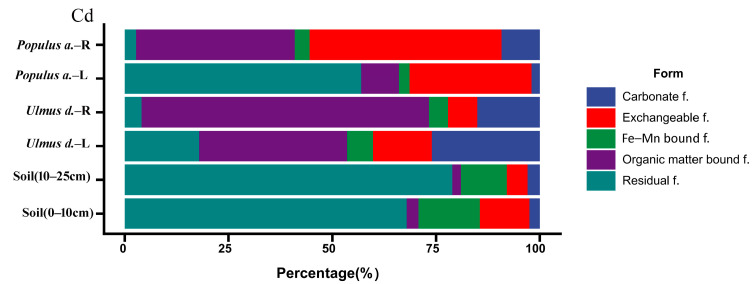
The stratified distribution of cadmium (Cd) speciation fractions.

The residual fraction (Residual f.) constituted the predominant Cd form in soils (60–80%), indicating Cd primarily existed in stable, insoluble phases. Notably, the exchangeable fraction (Exchangeable f.) exhibited maximum bioavailability, accounting for 35–52% of total Cd in plant roots—4–7 times higher than soil levels, demonstrating enhanced bioavailability. A unique organic matter-bound fraction (Organic matter-bound f.) enrichment was observed in *Ulmus* leaves (69.25%), revealing species-specific sequestration mechanisms. Interspecific variation in Cd speciation transformation was pronounced: Chenopodiaceae species preferentially accumulated exchangeable Cd, whereas *Ulmus* foliage showed organic complexation dominance. Vertical stratification analysis revealed elevated exchangeable Cd proportions in surface soils (0–10 cm: 11.9%) compared to deeper layers (5.3%), suggesting potential downward migration risks. These findings systematically characterize Cd fractionation dynamics and provide critical insights for ecological risk assessment and phytoremediation strategy optimization (Figure 5).

Residual form: Exhibited the highest average proportion (43.9%), representing the dominant Cd speciation in soils. Notably reduced in plant roots (minimum 2.7%), indicating limited bioavailability.

Organic matter-bound form: Displayed the largest coefficient of variation (CV = 1.14), reflecting substantial heterogeneity between soil and plant matrices. Reached 69.2% in elm roots, suggesting strong organic complexation capacity in this species.

Exchangeable form: Dominated in poplar roots (46.2%), implying high bioavailability and potential plant uptake efficiency.

*Ulmus pumila* preferentially accumulated the organic matter-bound fraction (69.3%), while *Populus* roots predominantly retained the exchangeable fraction (46.2%). This divergence implies species-specific Cd absorption mechanisms.

Soil-plant translocation dynamics: The residual fraction constituted 80.1% of total soil Cd but decreased markedly in plants (minimum 2.7%), demonstrating preferential plant assimilation of labile Cd forms over geochemically stable species.

Carbonate-bound form: Showed the second-highest variability (CV = 1.04) (Figure 5). Anomalously elevated in elm leaves (26.0%), potentially indicative of foliar adsorption from atmospheric deposition.

### 3.3. Compartment-Specific Dynamics of Cd Partitioning: Phytoaccumulation Gradients

**Figure 6:**
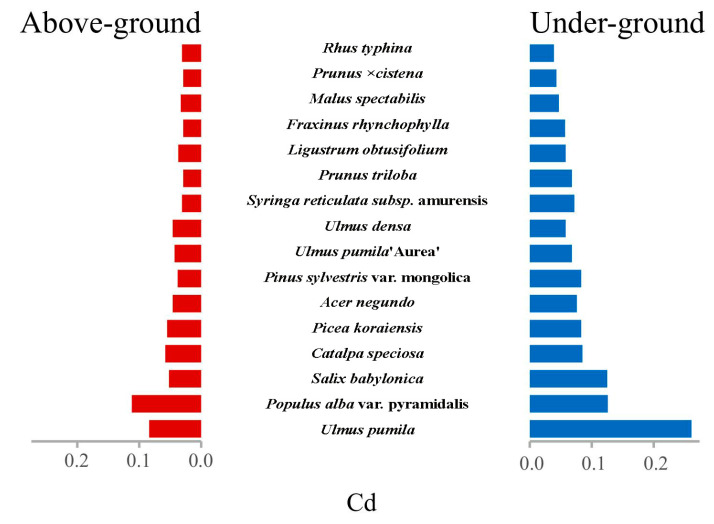
Cd accumulation between above-ground and below-ground compartments.

This reveals significant differences in Cd accumulation between above-ground and under-ground compartments (Wilcoxon *p* = 0.021*): *Ulmus pumila* showed the highest root-to-shoot translocation with BCF = 3.11 ± 0.89 (Mean ± SD), indicating strong phytoextraction potential. *Malus spectabilis* exhibited moderate accumulation (BCF = 1.42 ± 0.15), while *Ulmus pumila* demonstrated limited translocation (BCF = 1.26 ± 0.08). Below-ground Cd content was consistently higher across species (*p* < 0.05), with *Ulmus pumila* roots accumulating 0.261 mg/kg—3.1× higher than shoots (Figure 6).

Kruskal–Wallis test revealed marginally non-significant differences among species (*p* = 0.212), though notable trends emerged. *Ulmus pumila* had the highest absolute Cd accumulation in roots (0.261 mg/kg), significantly exceeding other species (*p* < 0.05 in post hoc Dunn’s test). *Pinus sylvestris* showed the lowest shoot accumulation (0.038 mg/kg), suggesting exclusion strategy. Coefficient of variation was highest in *Ulmus pumila* (CV = 29.8%), indicating environmental sensitivity.

*Ulmus densa*’s high BCF and root accumulation make it suitable for Cd-contaminated sites. Soil stabilization: *Malus spectabilis*’ balanced accumulation (BCF ≈ 1.4) suggests potential for phytostabilization. *Pinus sylvestris* may serve as buffer species in low-contamination zones.

*Ulmus pumila* exhibits elevated bioaccumulation capacity (BCF = 2.7 ± 0.3) with pronounced root-to-soil concentration gradients (7.4:1), demonstrating phytoextraction potential in heavily contaminated matrices. In contrast, *Malus spectabilis* maintains equilibrium between aerial and subterranean metal partitioning (BCF = 1.42 ± 0.15, root-shoot ratio = 0.93), indicative of rhizofiltration suitability for in situ stabilization. *Pinus sylvestris* shows differential compartmentalization patterns (BCF = 0.68 ± 0.09, leaf–root = 0.31), suggesting its ecological buffer function in marginal contamination zones through excluder-type allocation mechanisms.

Spinach (*Spinacia oleracea*) exhibited an exceptionally high Cd accumulation capacity (317.3 mg/kg), far surpassing other species. Bryophytes (*Thuidium tamariscinum*) and woody plants (*Ligustrum* spp.) showed minimal accumulation (<1 mg/kg), while rice (*Oryza sativa*) demonstrated significantly higher Cd concentrations (30.1 mg/kg) compared to common agricultural crops. The lichen *Pyxine cocoes* displayed moderate accumulation (16.9 mg/kg). Soil Cd levels in Urumqi (0.34–0.36 mg/kg) were markedly lower than those in Egyptian agricultural soils (7.84 mg/kg), reflecting distinct regional contamination profiles (Table 2).

## 4. Discussion

The patterns of heavy metal accumulation in specific organs of urban roadside trees reveal a hierarchical distribution, with bark containing the highest total metal content, followed by roots, branches, and leaves [39,40]. This hierarchy is consistent with previous research, which attributes the bark’s role as the primary interface for pollution to its structural and biochemical characteristics, including lipophilic polymers that are rich in metal-binding functional groups and a rough surface that facilitates particulate entrapment [41,42]. Additionally, the presence of senescent epidermal cell layers contributes to the progressive sequestration of metals over time [43]. Root systems exhibit secondary dominance through two mechanisms: passive uptake during nutrient absorption and active immobilization via cell wall passivation and compartmentalization, which localizes 60–75% of absorbed metals within rhizospheric regions [44,45,46]. Comparative analyses with urban flora further validate the general pattern of metal accumulation, with bark consistently exhibiting higher levels than branches and trunks, thereby reinforcing its efficacy in pollution interception [47,48].

The necessity for species-specific remediation strategies is highlighted by the varying capacities for Cd assimilation [49]. For example, *Ulmus densa* demonstrates a high root-to-leaf Cd ratio (6.37) and a preference for the uptake of organic-bound Cd (69.3%), positioning it as an effective phytoextractor. In contrast, *Malus spectabilis* (with a bioconcentration factor of approximately 1.4) and *Pinus sylvestris* are more adept at soil stabilization and delineating pollution boundaries, respectively [50,51]. These findings, along with the observed reduction in residual soil Cd from 80.1% to 2.7% in plant tissues, underscore the importance of species-specific selection in phytoremediation initiatives [52].

The significant differences observed in the chemical fractionation of Cd among various tree species (for instance, the dominance of organic-bound Cd in *Ulmus densa* compared to exchangeable Cd in Populus) are interpreted as being primarily influenced by species-specific rhizospheric processes. To enhance the separation of plant-induced effects from the natural variability of soil in phytoremediation research, it is essential for future studies to focus on the characterization of critical soil parameters (such as lutum, CaCO3, particle size distribution, organic matter content, and carbonate levels) prior to planting across experimental plots. Establishing this foundational data would facilitate a clearer attribution of the observed alterations in metal speciation to processes occurring in the rhizosphere.

## 5. Conclusions

This research reveals the mechanisms of organ-specific cadmium (Cd) accumulation and translocation in woody plants, emphasizing the role of roots as primary sinks and bark as significant secondary reservoirs.

Species-specific strategies such as hyperaccumulation, exclusion, and intermediate translocation facilitate customized phytoremediation applications. *Ulmus pumila* and *Pinus sylvestris* are particularly suitable for phytoextraction, whereas *Malus spectabilis* is effective for marking pollution boundaries. The strong correlation between roots and bark (r = 0.72) and the negative association between stems and leaves indicate a coordinated approach to transport and compartmentalization.

Data on Cd speciation demonstrate a preference for plant uptake of bioavailable fractions while residual forms are predominant in soils. These findings enhance the understanding of Cd biogeochemistry in trees and provide valuable insights for ecological restoration, pollution monitoring, and improved phytomanagement in contaminated areas.

## Figures and Tables

**Table 1:** Growth status of each greening tree species in the sampling area (Mean ± SD).

The Name of the Plant	The Average Tree Height H/m	Diameter at Breast Height D/cm	The Crown m
*Malus spectabilis*	5.43 ± 0.21	17.50 ± 0.41	5.20 × 5.70
*Ulmus pumila*	12.67 ± 1.25	18.50 ± 1.08	5.40 × 6.50
*Ulmus densa*	6.90 ± 0.41	16.17 ± 1.03	6.20 × 6.80
*Pinus sylvestris* var. mongolica	8.53 ± 0.82	13.33 ± 1.25	7.40 × 5.80
*Salix babylonica*	8.87 ± 0.39	25.67 ± 1.25	6.80 × 7.10
*Rhus typhina*	6.00 ± 0.51	11.57 ± 0.42	3.30 × 4.60
*Catalpa speciosa*	7.93 ± 0.50	13.67 ± 1.25	4.30 × 4.70
*Ulmus pumila* ‘Aurea’	6.33 ± 0.46	13.00 ± 0.82	3.50 × 4.60
*Ligustrum obtusifolium*	0.80 ± 0.29	/	/
*Fraxinus rhynchophylla*	7.90 ± 0.37	11.33 ± 1.26	4.50 × 5.80
*Prunus triloba*	1.10 ± 0.29	/	/
*Populus alba* var. pyramidalis	16.47 ± 1.13	18.33 ± 1.24	4.20 × 4.80
*Prunus × cistena*	1.20 ± 0.33	/	/
*Syringa reticulata* subsp. amurensis	7.73 ± 0.26	16.00 ± 0.82	3.50 × 4.40
*Acer negundo*	8.50 ± 0.65	16.13 ± 0.84	4.60 × 5.40
*Picea koraiensis*	5.67 ± 0.25	11.40 ± 0.98	3.70 × 4.60

Note: “/” means that the tree species is a small shrub, DBH and crown width can be ignored.

**Table 2:** Comparison of Cd concentrations reported in different literates.

Metal	Adsorption or Bio-Accumulation Status	Concentration(mg/kg)	Reference
Cd	*Spinach*	317.3	[32,33]
Cd	*Rice*	30.1	[34]
Cd	*Lettuce*	1.7	[32]
Cd	*Pyxine cocoes*	16.9	[35]
Cd	*Thuidium tamariscinum*	0.44	[36]
Cd	*Sphagnum papillosum*	4.31	[37]
Cd	Soil in Egypt	7.84	[38]
Cd	*Ulmus densa*	0.36	Urumqi, in this article
Cd	*Ligustrum obtusifolium*	0.08	Urumqi, in this article
Cd	Soil	0.34	Urumqi, in this article

## Data Availability

The original contributions presented in this study are included in the article/Supplementary Material. Further inquiries can be directed to the corresponding author.

## Supplementary Materials

The following supporting information can be downloaded at: https://www.mdpi.com/article/10.3390/toxics14020135/s1, Text S1. The description ICP-OES analysis; Text S2: Detailed information for the extraction method for heavy metal with different forms.
