# Supplementary material for: Comparative Analysis of Cadmium Accumulation in Xerophytic Plants: Implications for Species Selection in Phytoremediation
Source: Toxics. 2026 Jan 29;14(2):135. doi: 10.3390/toxics14020135 (PMC12944430; doi:10.3390/toxics14020135)
Supplement: Supplementary file 1 [file toxics-14-00135-s001.zip › toxics-4092874-supplementary.pdf]

Text S1. The description ICP-OES analysis

The ICP-OES analysis, when employed for elemental determination, operates under standardized conditions including an RF power of 1200-1500 W, plasma gas flow rate of 12-15 L/min, and auxiliary gas flow of 0.5-1.0 L/min, utilizing axial or radial viewing modes depending on element sensitivity requirements. Sample introduction typically involves a concentric nebulizer with cyclonic spray chamber maintained at 2-4°C, coupled with multi-element calibration standards (0.1-100 mg/L) to achieve detection limits in the ug/L range and linear calibration curves ($R^2 > 0.999$) for quantitative analysis, with quality control ensured through certified reference materials and periodic wavelength recalibration to maintain analytical precision.

Text S2. Detailed information for the extraction method for heavy metal with different forms

Exchangeable form: Measured 1 g of soil sample, mixed with 1.0 mol/L $MgCl_2$ solution at a ratio of 8:1, and extract at 25°C for 2 h. After centrifugation, dilute 5 mL of the supernatant to 50 mL and determined the element content. The soil samples underwent a wash with deionized water.

Carbonate form: Mix 1.0 mol/L NaAc-HAc solution (pH=7.0) with the residue at a ratio of 8:1 and extract for 2 hours at 25°C with constant temperature and vibration. Spined the supernatant in a centrifuge and take 5 mL, then diluted it to 50 mL to analyze the element content. Deionized water was used to wash the soil samples.

The Fe-Mn bound form: In the aforementioned residue, incorporate 0.04 mol/L NH_2OH HC1-4.5 mol/L HAc according to a liquid-soil ratio of 20:1. The content of each element was determined by diluting 5 mL of the centrifuged supernatant to 50 mL after intermittent vibration extraction in a constant temperature water bath at (96 ± 3) °C for 3 hours. The soil samples underwent washing with deionized water.

Organic matter bound form: In accordance with the liquid to soil ratio, introduced 0.02 mol/L HNO_3 and H_2O_2 (5:1) to the residue. Shaken intermittently for 2 hours at (85 ± 2)°C. Followed up with the addition of H_2O_2 solution (3:1) and continued shaking for 3 hours at (85 ± 2) °C. After cooling, introduced 3.2 mol/L HN_4Ac-20% HNO_3 in a 5:1 ratio, diluted to 100 mL, shaken for 0.5 mL, and added 3.2 mol/L HN_4Ac-20% HNO_3 in a 5:1 ratio, followed by HN_4Ac-20% HNO_3 in a 5:1 ratio, diluted to 100 mL. Following the cooling process, introduced (5:1) 3.2 mol/L HN_4Ac-20% HNO_3, diluted to 100 mL, and shaken for 0.5 h. Centrifuged the supernatant, reducing its volume to 5 mL, and then diluted it to 50 mL to measure the elemental content. Cleaned the soil sample by washing it with deionized water.

Residual form: In Method 1, add 0.10 g of dried residue to the digestion tank along with 5 mL of concentrated nitric acid and let it sit overnight. Combined 2 mL of hydrogen peroxide with 2 mL of hydrofluoric acid, then seal the stainless-steel rind. Placed it in the constant temperature drying box for ablation at 160 °C for 9 hours. Once cooled, removed it. The stainless-steel outer layer is gradually loosened as the inner canister is burned away. Heated the hot plate to 160°C and leaved it for 30 minutes. Next, 1% nitric acid was added in a volume of 25 mL. The purpose of the machine was to analyze the element composition.

Determination of potential trace element classification in plant samples (Xu, 2011): Fresh plant samples (0.5 g) were pre-weighed, cut into pieces, and grounded with 20 mL of extractant in a mortar and pestle. The resulting homogenate was then transferred to a 50 ml plastic centrifuge tube. The supernatant was extracted by oscillating at a constant temperature of 25 °C, followed by centrifugation (5000xg, 10 min) after 18 hours to recover the supernatant. An equal amount of fresh extractant was added to extract the samples again, which was then replaced every 2 hours. The process was repeated thrice, and the supernatant from all three repetitions was transferred into 150 mL triangular flasks. It was then subjected to extraction using five different extractants, in the following order of increasing polarity: 80% ethanol, deionized water, 1 M NaCl, 2% acetic acid, and 0.6 M hydrochloric acid.
